# Effects of Dietary Omega-3 Enrichment on the Chemical Composition and the Pathogenic Microbiota of Ovine Milk

**DOI:** 10.3390/foods11223736

**Published:** 2022-11-21

**Authors:** Athina Tzora, Chrysoula (Chrysa) Voidarou, Ilias Giannenas, Eleftherios Bonos, Konstantina Fotou, Aikaterini Nelli, Katerina Grigoriadou, Achilleas Karamoutsios, Zoitsa Basdagianni, Stella Dokou, Anastasios Tsinas, Ioannis Skoufos

**Affiliations:** 1 Laboratory of Animal Health, Food Hygiene and Quality, Department of Agriculture, University of Ioannina, 471 32 Arta, Greece; 2 Laboratory of Animal Nutrition, School of Veterinary Medicine, Faculty of Health Sciences, Aristotle University of Thessaloniki, 541 24 Thessaloniki, Greece; 3 Institute of Plant Breeding and Genetic Resources, Hellenic Agricultural Organization—DEMETER, Thermi, 570 01 Thessaloniki, Greece; 4 Laboratory of Animal Husbandry, Department of Animal Production, School of Agriculture, Aristotle University of Thessaloniki, 541 24 Thessaloniki, Greece

**Keywords:** ovine milk, ω-3 enriched diets, chemical composition, microbiota

## Abstract

The demand for ovine milk and ovine dairy products is constantly increasing due to their exceptional sensorial characteristics and their health benefits for consumers. However, dairy fat content and composition are of particular concern for consumers as well as the medical community, as there are risk factors for coronary disease, diabetes mellitus, cancer, and other serious diseases. For this reason, attempts have been made to control/regulate the fat composition of ovine milk by modifying sheep dietary intake of polyunsaturated fatty acids. In this experimental trial, a group of sheep were fed for 30 days a diet enriched in flaxseeds and lupines, feed ingredients rich in omega-3 fatty acids, aiming to investigate the effects on fat composition and the microbiota of ovine milk. Chemical analysis of the collected milk showed that the omega-3 and omega-6 content was increased. On the opposite, the atherogenic and thrombogenic indexes decreased. Of importance was the semi-protective effect on the udder by the increased omega-3 dietary intake, as depicted by its impact on the biodiversity of the pathogenic microbiota. These findings suggest that ovine milk could be modified under specific conditions to be more appropriate for the consumption by people belonging to high-risk groups for various diseases.

## 1. Introduction

Milk is essential for the development of the newborns of all mammals [1,2]. It is the only nutriment the newborns have for a certain period with its time length, which varies depending on the species [2,3,4]. Hence, the complexity of its chemical matrix reflects the necessity of the newborn to be provided with all the essential macro and micronutrients for its growth. Fats are such macronutrients with particular interest. To start, they consist of a variety of short-, medium-, and long-chained fatty acids (FAs) which can be classified according to their carbon skeleton length to a short chain, a medium chain, and a long chain FAs, or the abundance, location, and saturation level of their double or triple chemical bonds to saturated FAs or to mono- or poly-unsaturated FAs [2,5,6]. The FA variety is linked to their multiple physiological roles [2,7,8]. Apart from their primary role as energy sources, fats additionally act as biomolecules involved in various processes such as brain development, the function of the immune system, and the integrity of the cellular membranes [7,8,9,10,11].

Milk consumption is not limited to newborns. Many adults still enjoy consuming a variety of milk (skimmed milk, chocolate milk, or milk with various flavors), and an impressive spectrum of dairy products (fermented milk such as kefir, cheeses, butter, creams, and many others) are also available. The increased incidence of severe conditions and diseases such as obesity, cancer, diabetes mellitus, and coronary heart disease denounced many saturated FAs as detrimental to human health [12,13,14]. These warnings did not target exclusively dairy products, but their fats were included in the daily calculations of fat intake. Studies have revealed the beneficial and protective role of polyunsaturated FAs and, in particular, the omega-3 group. Numerous research papers have presented results of clinical studies showing that a high dietary intake of omega-3 FA exerts a protective role against various ailments of metabolic origin [15,16,17]. These findings were extensively communicated to the public resulting in increasing pressures which, in turn, forced the dairy industry to develop products rich in omega-3 FAs. Indexes, such as the atheromatic and the omega FA ratio, were implemented to assess the potential protective or detrimental effects of food products [16,17,18].

The possibility of manipulating the milk content of omega-3 FAs has become an interesting research topic [19,20,21,22] with obvious practical consequences. The research has focused on bovine milk due to its production in higher quantities in comparison to ovine and caprine milk [22,23,24]. Nevertheless, an increasing trend in the consumption of small ruminants’ milk and dairy products has been observed in recent years driving researchers’ interest in these types of milk [20,21,25]. The dairy industry uses the produced milk predominantly for cheese making [26]. It has been estimated that a fraction of 11% of the fat content of sheep milk consists of short- and medium-chain FAs, such as butyric acid (C4:0), which are beneficial to human health. Ovine milk has also a higher concentration of omega-3 FAs and conjugated linoleic acid (CLA) in comparison to the other ruminants’ milk [27], with the latter being involved in the regeneration of the nervous system [28]. Rumenic acid (cis-9, trans-11octadecadienoic acid) is considered the main isomer of CLA in the FAs fraction of ruminants’ milk [29].

**Figure 1:**
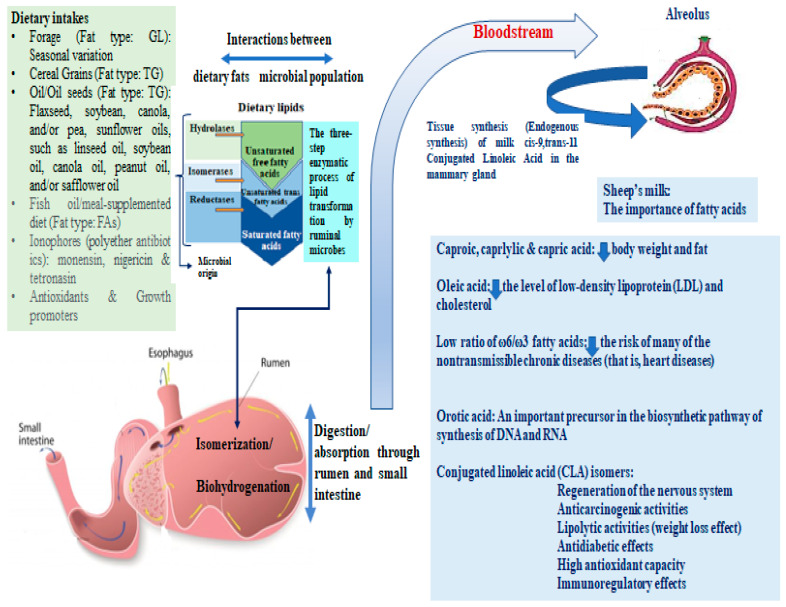
Sheep’s milk; Diagram of the dietary factors affecting ruminant fats, the three enzymatic steps of lipid transformation by ruminal microbes, the biosynthesis FA, and the importance of FAs to human health. TG: Triacylglycerides; GL: Glycolipids; FAs: Fatty acids.

Sheep milk is also rich in oleic acid (C18:1n9) which reduces the level of low-density lipoprotein and cholesterol [30,31]. Ovine milk’s omega-6 to omega-3 ratio (ω6/ω3) is 4.4 according to Luruena-Martinez et al. [32], lower than the respective ratio of the caprine milk [33]. Lower values of the omega-6/omega-3 ratio are associated with a lower risk of various cardiological conditions [34]. Figure 1 shows an interaction diagram between the dietary factors affecting the fat content in ruminants’ milk, the enzymatic transformation of lipids by ruminal microbiota, the mechanism of FA biosynthesis, and the impact of milk’s FAs on human health, simultaneously.

The ultimate question is how to retain and, if possible, increase the nutritional and health advantages of ovine milk while simultaneously reducing the risk factors associated with some of its components. So far, most researchers attempted to increase the omega-3 content in the ovine milk by increasing the dietary intake of these substances by sheep, but few papers, if any, dealt with the possibility of other parameters being affected by this feeding scheme, such as the health of the udder and the pathogenic microbiota of the milk. These topics should not be overlooked, because it is well documented that qualitative and quantitative changes in the diet might affect the chemical composition of the milk as well as its microbiota and the health of the udder.

The aim of this study is to bridge this experimental gap by investigating the effect of the increased dietary intake of omega-3 FAs on the FA content of ovine milk, as well as its correlation to the health of the udder, to the pathogenic microbiota and the chemical composition of the ovine milk.

## 2. Materials and Methods

### 2.1. Study Area

#### 2.1.1. Animals, Experimental Diets, and Management

The experiment was conducted on a sheep-rearing farm (Vonitsa, Preveza, Greece). Thirty crossbred lactating ewes (1/2 Lesvos breed and 1/2 Chios breed) at the early lactating period (producing 2.420 ± 0.23 kg milk per day), with a similar body live weight (averaging 55 ± 2.6 kg), and a similar age (second parturition, 24-month-old on average), were selected and used in a 60-day trial. In order to avoid potential variations of farm management and to strengthen the influences of the provided diet, the ewes were kept in a specially constructed area, isolated from the rest of the farming animals throughout the study. For the first 30 days, the selected ewes were fed a conventional diet (control diet), based on alfalfa hay, straw, and concentrate feed that contained soybean meal (days 1–30 of the trial). Then, beginning on day 31 of the trial and for a period of 1 month (days 31–60 of the trial), the selected ewes were fed a different concentrate feed with 50% less soybean meal that was equally substituted by flaxseed and lupins (experimental diet). The two diets were designed to be isonitrogenous and isocaloric. The chemical composition of the ingredients was based on the Premier Nutrition database [35]. The two diets are shown in Table 1.

#### 2.1.2. Milk Collection

The milk samples were collected from each ewe, from two consecutive milking periods (morning and evening) on the last day of the first trial period (day 30) and the last day of the second trial period (day 60). Emphasis was given on aseptic sampling during milk collection; therefore, teats were carefully cleaned and disinfected using disposable towels embedded with chlorhexidine, foremilk was stripped, and about 60 mL of milk was collected aseptically from each half-udder into sterile vials.

#### 2.1.3. Blood Sample Collection

On days 30 and 60 of the trial, blood samples were collected from the jugular vein of each sheep 3 h after the morning feeding and centrifuged for 10 min at 3000 rpm at 25 °C to separate serum, which was then stored at −20 °C for a subsequent serum biochemical analysis.

### 2.2. Microbiological Analysis

An aliquot of each milk sample (1 mL) and serial of 10-fold dilutions were prepared using tubes containing 9 mL of sterile 0.1% buffer peptone water. Subsequently, 0.1 mL of each sample and dilution were cultured into Petri dishes with Nutrient agar (Oxoid Ltd., Basingstoke, UK), MacConkey agar (Merck, Darmstadt, Germany), 5.0% Sheep Blood agar [SBA, (Becton Dickinson, Sparks, NV, USA)], Chocolate agar (Oxoid Ltd., Basingstoke, UK), Bismuth Sulphite agar (Merck, Darmstadt, Germany) and Phenol Red Brilliant Green agar (Merck, Darmstadt, Germany), and incubated aerobically as well as anaerobically, for 24 to 72 h at 37 °C. The PALCAM agar (Oxoid Ltd., Basingstoke, UK) was also used after enrichment in a Buffered Listeria enrichment broth at 30 °C for 48 h and, subsequently, the Petri dishes were incubated at 37 °C for 72 h.

Furthermore, for the detection of *Staphylococcus* spp., the following scheme was conducted [36,37]: 1 mL of each milk sample was enriched in 9 mL of Mueller–Hinton broth supplemented with 6.5% NaCl (Oxoid Ltd., Basingstoke, UK). Another 1 mL was enriched in 9 mL of Tryptone Soya broth with 10% NaCl and 1% sodium pyruvate (HiMedia Labs, Einhausen, Germany). Subsequently, the aforementioned broths were incubated at 37 °C and 35 °C, respectively, for 20 h and followed by incubation onto Baird-Parker agar (Oxoid Ltd., Basingstoke, UK) containing 30% egg yolk with 1% tellurite (Oxoid Ltd., Basingstoke, UK) and mannitol salt phenol red agar (Merck, Darmstadt, Germany) plates.

All isolates were further characterized according to their registered protein and peptide information analysis results using the Microflex LT MALDI-TOF MS (matrix-assisted laser desorption ionization time-of-flight mass spectrometry) (Bruker Daltonics, GmbH, Bremen, Germany).

#### Identification of Microbiota by the MALDI-TOF MS

The bacterial isolates’ identification was performed with the Bruker Microflex™ LT MALDI-TOF MS system (Bruker Daltonik GmbH, Bremen, Germany) according to the ethanol-formic acid extraction procedure, as previously described [38,39]. Acquisition and mass spectra analyses were performed by the MALDI Biotyper software package (MBT Compass version 4.1) and the reference database version (BDAL Rev. No 11, 10,833 database entries) (Bruker Daltonik GmbH, Bremen, Germany). A Bruker’s Bacterial Test Standard (Bruker Daltonik GmbH, Bremen, Germany) was used for the analysis calibration according to the manufacturer’s instructions. Τhe MALDI-TOF MS Biotyper software compares each isolates’ mass spectrum to the database reference strains’ mass spectra calculating a random unit score value between 0 and 3, determining the similarity between the analyzed sample and the reference spectrum. Score values of ≥2.0 were accepted for species assignment, scores of ≥1.7 but <2.0 for identification to the genus level, and scores below 1.7 were considered unreliable. Only isolates with a of score ≥2.0 were used in the current study.

### 2.3. Analysis of Samples by the California Mastitis Test and Categories of the Somatic Cell Count

The sheep were examined clinically for swelling, the presence of lesions, or anatomical malformations, and milk from individual halves was evaluated by the California mastitis test (CMT). The CMT scores were 0, +, ++, and +++ for: “negative”, “weak positive”, “positive”, and “strong positive”, respectively [40,41].

### 2.4. Chemical Analysis/Biochemical Assay

#### 2.4.1. Milk Samples

Milk samples were analyzed for fat, protein, lactose, and total solids using an infrared method MilkoScan FT120 (FOSS Electric, Hillerød, Denmark) in transmittance mode. Samples were thawed at room temperature (20 °C) prior to their analysis. Milk pH was also measured by a portable pH meter Sentron 1001 (Sentron Europe, Roden, The Netherlands) [42]. Each milk sample was measured in triplicate.

##### Determination of the Milk Samples FA Composition

Fatty acid methyl esters (FAMEs) were assessed from the milk samples using the methods described by Bligh and Dyer [43] and the International Organization for Standardization (ISO) [44], as has been reported previously by Papaloukas et al. [45]. The fatty acid esters (FAMEs) were identified and quantified by an Agilent Technologies 6890N (Santa Clara, CA, USA) gas chromatograph (GC), equipped with a flame ionization detector (FID) and a DB-23 capillary column (60 m × 0.25 mm i.d., 0.25 μm film thickness). Each peak was identified and quantified as a percentage (%) of the total FA, using appropriate standards (“37 component FAME mix”, 47885-U, Supelco; “PUFA-No.2, Animal source”, 47015-U, Supelco). Then, the total saturated fatty acids (SFA), monounsaturated fatty acids (MUFA), polyunsaturated fatty acids (PUFA), unsaturated fatty acids (UFA), n-3 PUFA, and n-6 PUFA were calculated as the sums of the relevant individual FA.

#### 2.4.2. Blood Samples

Blood was centrifuged (Hettich Rotina 35R centrifuge (Andreas Hettich GmbH & Co. KG., Tuttlingen, Germany) at 3000 rpm for 10 min at 25 °C, prior to the analysis. Blood serum parameters including albumin, total cholesterol, glucose, aspartate aminotransferase (AST), alanine aminotransferase (ALT), and creatine kinase (CK), were measured using an auto-analyzer (VetTest 8008 serum chemistry analyzer and VetTest reagent slides, IDEXX Laboratories Inc., Westbrook, ME, USA) according to the manufacturer’s instructions.

### 2.5. Calculations of Health Indexes

The index of atherogenicity (AI) and the index of thrombogenicity (TI) were used to evaluate the nutritional quality of the fatty acid profile. The Index of Atherogenicity (AI) was estimated, according to Ulbricht and Southgate [46] and Paszczyk and Luczynska [47], as follows:

(1) $$\mathrm{AI}=\frac{\;C12:0+\left(4\times \;C14:0\right)+\;C16:0}{\;\Sigma n-3\;\mathrm{PUFA}\;+\;\Sigma n-6\;\mathrm{PUFA}\;+\;\Sigma \;\mathrm{MUFA}}$$

(2) $$\mathrm{TI}=\frac{C14:0+\;C16:0+\;C18:0}{\left(0.5\times \;C18:1\right)+\left(0.5\times \;\mathrm{other}\;\mathrm{MUFA}\right)+\left(0.5\times \;\Sigma n-6\;\mathrm{PUFA}\right)+\left(3\times \;\Sigma n-3\;\mathrm{PUFA}\right)+\frac{\Sigma n-3\;\mathrm{PUFA}}{\Sigma n-6\mathrm{PUFA}}\;}$$

Hypocholesterolemic fatty acids (DFA), hypercholesterolemic fatty acids (OFA), and the H/H index (DFA/OFA) were calculated according to Paszczyk and Luczynska [47] and Osmari et al. [48]:

(3) DFA=UFA+C18:0

(4) OFA=C12:0+C14:0+C16:0

(5) $$H/H\;\mathrm{index}=\frac{\mathrm{DFA}\;}{\mathrm{OFA}\;}$$

### 2.6. Statistical Analysis

The experiments were performed in triplicate, and the same pattern was followed for the analyses (performed in triplicate). The data for the raw milk samples were analyzed using the SPSS statistical package (Version 20, IBM SPSS). The general linear model test was used to compare the means of the two treatments (control vs. experimental). A Mann–Whitney test was applied for data that were not homogeneous. Pearson’s correlation coefficient was used to detect any possible correlations among the investigated parameters. A significance level was set at 5% (*p* < 0.05) for all tests.

## 3. Results

In the present study, thirty adult sheep were fed for 30 days a diet enriched in omega-3 by the addition of specific feed ingredients. Milk samples were taken from each animal the day before the 30-day period, as well as at the end of this period, and then they were compared for their FA content and other chemical parameters and for their pathogenic microbiota. The experimental design focused on the alteration of the diet as the sole factor of differentiation.

Table 2 presents the effects of dietary supplementation on milk production and related quality parameters. Milk fat percentage and total solid were significantly increased (*p* < 0.001) in the experimental diet compared to the control diet. The other examined parameters were not significantly affected (*p* > 0.05). Most milk samples showed values of CMT 0, with a few cases where a value of 1 was detected.

The effects of the dietary supplementation on the ovine milk fatty acid profile are shown in Table 3. The dietary supplementation with flaxseed and lupins increased C10:0 (*p* ≤ 0.05); decreased C14:1 (*p* ≤ 0.05); decreased C16:0 (*p* ≤ 0.05); decreased C18:0 (*p* ≤ 0.05); increased C18:2 ω9 cis (*p* ≤ 0.05); increased C18:2 v6 trans (*p* ≤ 0.001); increased C18:3 ω3 (*p* ≤ 0.001); decreased total saturated fatty acids (*p* ≤ 0.01); increased unsaturated fatty acids (*p* ≤ 0.05); increased the ratio of unsaturated/saturated fatty acids (*p* ≤ 0.05); increased total polyunsaturated fatty acids (*p* ≤ 0.001); increased total ω3 fatty acids (*p* ≤ 0.001); increased total ω6 fatty acids (*p* ≤ 0.01); decreased the ratio of ω6/ω3 fatty acids (*p* ≤ 0.01); decreased the atherogenicity index (*p* ≤ 0.05); and decreased the thrombogenic index (*p* ≤ 0.001). The other examined milk parameters did not differ between the treatments (*p* > 0.05). The dietary supplementation of flaxseed and lupins significantly affected the concentration of various FAs in the ovine milk. It is interesting to note that the FAs with an odd number of carbons, such as pentadecanoic (C15:1) and margaric (C17:0), were not affected. No effect was also observed on the very short-chain fatty acids (C4:0–C8:0).

The results of the ovine blood analysis are presented in Table 4. The dietary supplementation with flaxseed and lupins increased ovine blood and total cholesterol (*p* ≤ 0.001), whereas the other examined parameters were not affected (*p* > 0.05) by this supplementation.

Table 5 presents the differences in the prevalence of various bacterial strains isolated from the milk of sheep prior to and after feeding them with feeds enriched in omega-3 fatty acids. Overall, 30 and 26 isolates were recovered from the milk samples of the first and the second sampling, respectively. In total, sixteen species were isolated, seven from the first sampling and twelve from the second sampling. Three of them were common to both samplings, while four were isolated only in the first sampling (control period), and nine were isolated only in the second sampling (experimental period). Our results show that when the feeding practice changed, either some species disappeared, new species emerged, or strains of the same species were reisolated but in different animals than the ones which were isolated in the control period. *Corynebacterium stationis*, for example, was the most abundant microorganism and was isolated from nine animals’ milk in the control period (first sampling). However, the bacterium was not reisolated in milk from four of the nine animals fed the enriched in omega-3 fatty acids diet and was isolated in two other animals whose milk was not isolated prior to feeding the enriched diet (second sampling). A similar effect was observed for *Staphylococcus xylosus* and *S. epidermidis*. Species such as *S. chromogenes*, *S. auricularis*, and *S. petrasii* were not isolated from the second sampling (omega-3 fed), while species such as *S. haemolyticus*, *S. caprae*, and Enterobacteriaceae were not isolated from the first sampling (control).

Coagulase-negative staphylococci (CNS) are of particular interest due to their importance as the main causative agent for subclinical mastitis. Five species of CNS were isolated from the first sampling (twenty isolates in total) and four species were isolated from the second sampling (twelve isolates in total). These findings show that the microbiota of the milk was significantly affected by the changes in the sheep diet.

## 4. Discussion

Ovine milk is very popular in many countries worldwide, particularly, in the Mediterranean area and Asia, and is growing in the European and USA markets. It is a significant source of FAs, vitamins, calcium, phosphorus, iron, and magnesium. Various efforts have been made to change its fat content in a healthier direction, which is to lower the hazardous FAs and to increase the beneficial ones, such as the omega-3 [5,30,49,50]. The CLA isomers contained in sheep milk may induce beneficial effects on human health, such as “anti-cancer” properties, lower triglyceride concentrations, and reduced blood cholesterol and glucose levels [5,51,52]. These isomers also normalize the functions of the immune system [53,54]. The cis-9, trans-11 CLA, and trans-10, cis-12 CLA isomers affect specific T cell populations and immunoglobulin subclasses.

Small ruminant breeding for milk production is a growing field and simultaneously constitutes a diachronic tradition for countries, such as Greece, where it is of great significance for the growth of the primary sector. The increased content of proteins and, in particular, casein, fats, and calcium in ovine milk make it an exceptional medium for cheese production [55,56], It is for this reason that a variety of cheeses with outstanding organoleptic characteristics is produced by ovine milk [57]. The chemical composition of ovine milk is responsible for its superior nutritional value and higher caloric content compared to bovine and caprine milk [56,57,58]. Sheep milk is rich in short- and medium-chain saturated fatty acids such as caproic acid (C6:0), caprylic acid (C8:0), and capric acid (10:0). However, the content of other saturated FAs, such as butyric acid, is low [30]. The former three FAs play a crucial role in decreasing body weight and body fat percentage [5,30]. These FAs are hydrolyzed from the triglycerides and are directly transferred to the liver following absorption where they are utilized as readily available energy sources, properties extremely useful in clinical cases, such as malnutrition or malabsorption syndrome [59].

The findings of the present trial are in accordance with Caroprese et al. [60] and were in a sense anticipated since numerous studies have demonstrated that in dairy ewes, the energy content of the diet is the most important factor manipulating the milk fat content and yield [61].

Milk’s PUFA omega-3 content was increased by a factor of 4.3, while PUFAs in omega-6 increased by a factor of 1.6. These changes result in a decrease in the omega-6/omega-3 ratio by a factor of almost three. In order to investigate any possible correlations among the fatty acids in each sampling, as well as between FA in the two different samplings, Pearson’s correlation coefficients were calculated for every FA between the two samplings (data not shown). Nearly all values of Pearson’s coefficients (except in two cases which will be immediately described) revealed no significant correlations. This finding implies that there must be a strong idiosyncratic element in the animals involved concerning the composition of the fat content of their milk, given the fact that all the conditions of the experiment were identical for all animals. The verification of this suggestion by further research opens the possibility for selective breeding focused on animals with specific characteristics in their milk fat composition resulting in ovine milk with higher omega -3 concentrations. In both samplings corresponding to the two treatments (with and without dietary omega-3 supplementation), the total saturated (SFA) and the total unsaturated (UFA) FAs were strongly negatively correlated (a Pearson’s correlation coefficient of R = −0.993 and R = −0.9957, respectively, *p* < 0.001). The significant increase in the total fat content of the milk after the dietary intake of feed ingredients rich in omega-3 FAs (second sampling, Table 2) did not affect this correlation, suggesting antagonistic metabolic pathways. The thrombogenic and atherogenic indexes were not significantly correlated in the control milk samples but they were strongly positively correlated in the experimental milk samples (R = 0.9412, *p* < 0.001), possibly due to the increase of MUFAs and PUFAs. Both these indexes were reduced in the control samples because of increased denominators following the increase in UFAs, in PUFAs-ω3, and in PUFAs-ω6.

Regarding the blood analysis, from the examined parameters, only the cholesterol levels were significantly increased (*p* < 0.001), indicating that the omega-3 FAs were absorbed in the intestine and directly delivered via the blood circulation to the mammary gland.

The chemical composition of milk, in terms of fat content and its FA composition, depends on dietary (composition and availability), animal (breed, lactation stage, and body condition), and environmental (especially cold and heat stress) factors [5,62]. In our study, these factors were identical for both samplings due to the experimental design. More than 98% of lipids in ovine milk have the typical structure of glycerol and three FAs with different carbon chain lengths. The FAs synthesized by the epithelial cells of the mammary glands and secreted in the milk are either delivered by blood plasma circulation, due to feed uptake, synthesized in the liver, or by de novo synthesis. The de novo synthesis of FAs by the cells of the mammary gland leads to the production of short-chain (C4:0–C14:0) fatty acids and a portion of C16:0 and stems from acetate and beta-hydroxybutyrate produced in the rumen by fermentations. Our findings show that the milk content of most of the small- and medium-chain fatty acids in both samplings was not affected by the experiment. However, the differences concerning the C10:0, the C14:1, and the C16:0 FAs, as well as the C18:0 FA, were significant and lead to proportional alterations in the total SFAs between the two samplings (Table 3). It seems that the increased dietary intake of omega-3 FAs affects the metabolism of fats in all the aforementioned levels and further research will reveal the exact mechanisms. It is possible, for example, that in some cases or under certain conditions, the de novo synthesis of FAs in the mammary gland cells could generate larger quantities of FAs with respect to the amount produced by the liver, or vice versa. These are still unresolved issues and further research is required. The rest part of C16:0 and the long-chain FAs (C18:0–C22:0) are derived from lipids originating from absorption in the small intestine or from utilization and decomposition of adipose tissue fat. The FAs with a chain length from 14 to 18 carbons are further processed in the mammary gland through the activity of desaturase enzymes. Thus, the observed increase of the C18:2 ω9 cis, the C18:2 ω6 trans, and the 18:3 ω3 in comparison to the C18:0 decrease can be attributed to antagonistic dominion over the same absorption route, since the intake of omega-3 fatty acids was dietarily increased, implying that a significant portion of them managed to pass through the rumen and evaded desaturation in the mammary gland. Finally, the odd and the branched FAs contained in milk are absorbed in the intestine and originate from the cell membrane of bacteria [63,64,65].

Supplementation of UFA diets may result mainly in the increase of MUFA, as well as PUFA, to a lesser extent, such as vaccenic acid (VA) and conjugated isomers of linoleic acids (CLA), respectively [33,66,67]. The higher concentration of these FAs in ewe’s milk is dependent on the composition of the diet consumed by the animals [68,69] which was the only factor differentiating the two samplings of the current experiment. The researchers in [70,71] state that up to 15% of the total C18:1 in sheep milk is trans configuration, and vaccenic acid was found to be the principal acid [72,73]. In the present study, although CLA was found to increase in the milk of the experimental group (Table 3), this difference was not statistically significant due to the high fluctuation of the values.

Sheep’s milk has a higher CLA content than goat’s milk due to differences in the mRNA of the adipocytes of their mammary glands [74]. The cis-9, trans-11 octadeca-dienoic acid (rumenic acid) is the major isomer of CLA present in the milk fat of ruminants (Table 3). Among the ruminants, sheep’s milk is the richest (1.1%) in CLA, though its concentration is season-dependent [5,30,59,75], perhaps due to seasonal variation of the grazing vegetation. In our study, the animals were grazing the same fields with the same controlled vegetation (species) in both samplings.

The two different diets (control vs. enriched) affected milk pathogenic bacteria diversity, taking into consideration that the animals were the same breed and continued under the same conditions of management (facilities, personnel, etc.). The results show a significant differentiation of the prevalence of the various isolated species of the pathogenic microbiota and, since no other factor was changed by the experimental design, it indicates that these differentiations must be attributed to the effect of the omega-3-rich diet. Pathogens such as *Corynebacterium stationis*, *Staphylococcus epidermidis*, and opportunistic pathogens such as *Staphylococcus xylosus*, were isolated less frequently after the omega-3 enrichment of the diet. Fewer new infections were recorded while more animals were completely free from these bacteria. In the case of *S. epidermidis*, from the initial eight animals’ milk samples, this bacterium was isolated from only one animal that retained the infection at the second sampling (day 60). Furthermore, the other three CNS species which were isolated from the first sampling, considered along with the previous two as the most common causative agents of subclinical mastitis, were not isolated in the second sampling; however, new species of CNS were isolated at this sampling time, indicating a moderate protective effect. *Manheimia haemolytica* is also a common pathogen causing serious bronchopneumonia in goats and sheep of all ages with high morbidity rates, particularly in kids and lambs [76,77,78]. These bacteria were not isolated from the second sampling. The species isolated in the second sampling are generally less virulent and less pathogenic for the udder, except, of course, for the *Staphylococcus aureus* and the *Staphylococcus haemolyticus*, which are involved in the pathogenesis of mastitis. *Streptococcus parasanguinus* is rarely pathogenic for the udder and the same holds true for *Streptococcus pluranimalium* [79] and for *Proteus mirabilis*, which is known to cause cystitis and pyelonephritis and is present in soil and water [80]. *Veillonella parvula* is not able to ferment carbohydrates and lives on the lactate produced by Streptococci [81,82], so it is reasonable to assume that this bacterium was rather isolated due to the isolation of the aforementioned Streptococci. Finally, the two Enterobacteriaceae species (*Enterobacter cloacae* and *Enterobacter kobei*), although pathogenic for the respiratory tract, are considered major pathogens for the mammary gland [83,84]. All these findings suggest a semi-protective effect of the dietary omega-3 fatty acids on bacterial populations in the udder. Semi-protective can be explained in the sense that the main milk pathogens present in the control group were significantly reduced; however, not all of them vanished from the udder. Additionally, the total pathogenic bacterial load was found to be diminished.

## 5. Conclusions

− The supplementation of feeds rich in omega-3 FAs for a 30-day period increased the ovine milk fat and total solids content.
− The milk’s FA profile was modified by the dietary omega-3 FA enrichment resulting in milk with increased amounts of UFA and improved atheromatic and thrombogenic indexes.
− The pathogenic microbiota of the ovine milk was affected by the dietary omega-3 FA enrichment. Most pathogens were reduced or even eliminated in the second sampling, i.e., after 30 days of flaxseed and lupin administration. The biodiversity in terms of pathogenics for the udder species were altered towards a healthier direction. However, some pathogens remained while some other species emerged. Thus, the total effect can be possibly characterized as semi-protective for the udder.
− These findings suggest that ovine milk could be beneficially modified by diets enriched in omega-3 FAs and could potentially be more appropriate for consumption by people belonging to high-risk groups for age-related and metabolic disorders.

## Figures and Tables

**Table 1:** Daily ingredient allowance and chemical composition of the diets offered to dairy ewes during the trial.

Ingredients (On Fresh Weight Basis)	Control Diet (g/Day/ewe)	Experimental Diet (g/Day/ewe)
Lucerne hay	1.200	1.200
Barley straw	300	300
Corn grain	540	590
Barley grain	330	150
Wheat bran	180	110
Sunflower seed meal (36% crude protein)	30	180
Soybean meal (47% crude protein)	300	150
Cotton seed	30	80
Flaxseed	-	75
Lupin seed	-	75
Molasses	20	20
Premix ^1^ containing vitamins and inorganic minerals	60	60
Total dry matter intake/day	2630	2640
**Chemical Analysis (%)**		
Dry matter	87.4	87.5
Crude protein (N × 6.25)	16.1	16.1
Ether extract	2.82	4.01
Neutral detergent fiber	33.8	35.1
Acid detergent fiber	19.5	19.9
Acid detergent lignin	4.51	4.98
Ash	6.55	6.52
Starch	18.0	16.1
**Calculated analysis**		
Calcium (g/kg)	11.5	11.6
Phosphorus (total) (g/kg)	3.82	3.98
PDI (g/kg DM)	89.4	87.5
PDIA (g/kg DM)	44.3	42.7
UFL	0.71	0.71
Total saturated fatty acids (%)	0.48	0.59
Total unsaturated fatty acids (%)	1.69	2.72
Omega-6 fatty acids (%)	0.99	1.15
Omega-3 fatty acids (%)	0.28	0.82
Omega-6/Omega-3	3.53	1.41

^1^ Vitamin and mineral mix contained per kg DM of concentrate: 8000 IU of vitamin A; 90 mg of vitamin E; 3000 IU of vitamin D3; 1.5 mg/kg biotin; 6 mg/kg niacin; 45 mg/kg choline; 0.2 mg Co; 3 mg I; 100 mg/kg Fe; 50 mg Mn; 0.45 mg Se; 150 mg Zn; 6 g of NaCl; 4 g of Sulfur; 10 g of magnesium oxide; 15 g of monocalcium phosphate, and 21 g of limestone. PDI = protein digestible in the small intestine, PDIA = protein digestible in the small intestine supplied by rumen—undegraded dietary protein, UFL = forage unit for lactation.

**Table 2:** Effect of dietary supplementation on ovine milk amount, total mesophilic microflora, and chemical composition.

Milk Analysis	Control Diet Day 30 of Trial	Experimental Diet Day 60 of Trial	SEM	*p*
Milk amount at morning milking (mL)	1375.0	1371.4	47.03	0.971
Milk total mesophilic flora (Log CFU/g)	4.119	3.808	0.1740	0.381
Milk fat (%)	3.85 ^a^	5.50 ^b^	0.203	<0.001
Milk protein (%)	4.59	4.66	0.071	0.648
Milk lactose (%)	5.36	5.43	0.035	0.277
Milk total solids (%)	14.57 ^a^	16.22 ^b^	0.223	<0.001
Milk solids nonfat (%)	10.62	10.76	0.072	0.359

^a, b^ Values in the same row with different superscripts differ significantly (*p* < 0.05).

**Table 3:** Effect of dietary supplementation on milk fatty acid profile.

Milk Fatty Acid Profile (% of Total Fatty Acids)	Control Diet Day 30 of Trial	Experimental Diet Day 60 of Trial	SEM	*p*
C4:0; Butyric	0.73	0.90	0.122	0.479
C6:0; Caproic	4.25	4.43	0.320	0.790
C8:0; Caprylic	4.43	4.51	0.242	0.867
C10:0; Capric	8.84 ^a^	11.47 ^b^	0.672	0.048
C12:0; Lauric	5.24	4.96	0.843	0.870
C14:0; Myristic	11.56	11.16	0.500	0.695
C14:1; Myristoleic	0.43 ^b^	0.29 ^a^	0.036	0.047
C15:0; Pentadecylic	1.15	1.15	0.084	0.985
C15:1; Pentadecenoic acid	0.12	0.10	0.021	0.650
C16:0; Palmitic	26.22 ^b^	22.92 ^a^	0.689	0.013
C16:1; Palmitoleic	0.99	0.82	0.098	0.405
C17:0; Margaric	0.72	0.58	0.071	0.346
C17:1 ω10 cis; Heptadecenoic acid	0.15	0.11	0.030	0.522
C18:0; Stearic	12.87 ^b^	9.51 ^a^	0.766	0.025
C18:1 ω9 trans; Elaidic	1.17	0.98	0.138	0.488
C18:1; ω11 trans; Vaccenic acid	2.09	2.29	0.273	0.725
C18:1 ω9 cis; Oleic	16.23 ^a^	18.56 ^b^	0.559	0.034
C18:2 trans; Conjugated linoleic acid	0.11	0.26	0.050	0.124
C18:2 ω6 trans; Linoelaidic	0.29 ^a^	1.83 ^b^	0.247	0.001
C18:2 ω6 cis; Linoleic	2.21	2.20	0.223	0.978
C18:3 ω3; Linolenic acid	0.17 ^a^	0.73 ^b^	0.065	<0.001
Short-chain fatty acids—SCFA	4.98	5.33	0.349	0.624
Medium-chain saturated fatty acids—MCFA	18.51	20.94	1.155	0.303
Long-chain saturated fatty acids—LCFA	76.47	73.47	1.318	0.265
Saturated fatty acids—SFA	76.00 ^b^	71.59 ^a^	0.889	0.010
Unsaturated fatty acids—UFA	23.95 ^b^	28.15 ^a^	0.862	0.011
UFA/SFA ratio	0.32 ^a^	0.40 ^b^	0.017	0.014
Monounsaturated fatty acids—MUFA	21.18	23.14	0.649	0.133
Polyunsaturated fatty acids—PUFA	2.78 ^a^	5.01 ^b^	0.349	<0.001
PUFA ω3	0.17 ^a^	0.73 ^b^	0.065	<0.001
PUFA ω6	2.50 ^a^	4.02 ^b^	0.287	0.005
ω6/ω3	17.32 ^b^	5.85 ^a^	2.047	0.003
Δ-9 dehydrogenase C14:1/C14:0	0.04	0.03	0.004	0.136
Δ-9 dehydrogenase C16:1/C16:0	0.04	0.04	0.004	0.923
Atherogenicity index (C12:0 + 4 × C14:0 + C16:0)/UFA	3.30 ^b^	2.67 ^a^	0.151	0.034
Thrombogenic index (C14:0 + C16:0 + C18:0)/(0.5 × MUFA + 0.5 × PUFAω6 + 3 × PUFAω3 + ω3/ω6)	4.11 ^b^	2.79 ^a^	0.176	<0.001

^a, b^ Values in the same row with different superscripts differ significantly (*p* < 0.05).

**Table 4:** Effect of dietary supplementation on ovine blood parameters.

Blood Parameters	Control Diet Day 30 of Trial	Experimental Diet Day 60 of Trial	SEM	*p*
Albumin—ALB (g/DL)	3.0	2.9	0.06	0.762
Alanine aminotransferase—ALT (U/L)	59.6	66.9	2.65	0.174
Aspartate aminotransferase—AST (U/L)	130.7	125.2	7.37	0.720
Glucose—GLU (mg/DL)	62.7	63.4	2.86	0.907
Cholesterol—CHOL (mg/DL)	44.1 ^a^	57.3 ^b^	2.20	0.001
Creatine kinase—CK (U/L)	74.9	81.9	3.55	0.337

^a, b^ Values in the same row with different superscripts differ significantly (*p* < 0.05).

**Table 5:** Bacterial isolates from the milk of sheep before (control diet—day 30 of trial) and after 30 days of feeding feeds enriched in omega-3 fatty acids (experimental diet—day 60 of trial).

Species	Control Diet Day 30 of Trial	Experimental Diet Day 60 of Trial	Isolated Only on Day 30	Isolated Both on Day 30 and Day 60	Isolated Only on Day 60
*Corynebacterium stationis*	9	7	4	5	2
*Staphylococcus xylosus*	8	4	6	2	2
*Staphylococcus epidermidis*	8	4	7	1	3
*Staphylococcus chromogenes*	2	-	-	-	-
*Staphylococcus auricularis*	1	-	-	--	-
*Staphylococcus petrasii*	1	-	-	-	-
*Staphylococcus haemolyticus*	-	3	-	-	-
*Staphylococcus aureus*	-	1	-	-	-
*Staphylococcus caprae*	-	1	-	-	-
*Manchemia haemolytica*	1	-	-	-	-
*Streptococcus pluranimalis*	-	1	-	-	-
*Streptococcus parasanguinus*	-	1	-	-	-
*Proteus mirabilis*	-	1	-	-	-
*Veillonella parvula*	-	1	-	-	-
*Enterobacter kobei*	-	1	-	-	-
*Enterobacter cloacae*	-	1	-	-	-

## Data Availability

Data are contained within the article.
